# *Dicer1* Depletion in Male Germ Cells Leads to Infertility Due to Cumulative Meiotic and Spermiogenic Defects

**DOI:** 10.1371/journal.pone.0025241

**Published:** 2011-10-05

**Authors:** Yannick Romero, Oliver Meikar, Marilena D. Papaioannou, Béatrice Conne, Corinne Grey, Manuela Weier, François Pralong, Bernard De Massy, Henrik Kaessmann, Jean-Dominique Vassalli, Noora Kotaja, Serge Nef

**Affiliations:** 1 Department of Genetic Medicine and Development, University of Geneva Medical School, University of Geneva, Geneva, Switzerland; 2 Department of Physiology, Institute of Biomedicine, University of Turku, Turku, Finland; 3 Institut de Génétique Humaine, IGH - CNRS, Montpellier, France; 4 Center for Integrative Genomics, University of Lausanne, Lausanne, Switzerland; 5 Department of Internal Medicine, University Hospital, Lausanne, Switzerland; Beckman Research Institute of the City of Hope, United States of America

## Abstract

**Background:**

Spermatogenesis is a complex biological process that requires a highly specialized control of gene expression. In the past decade, small non-coding RNAs have emerged as critical regulators of gene expression both at the transcriptional and post-transcriptional level. DICER1, an RNAse III endonuclease, is essential for the biogenesis of several classes of small RNAs, including microRNAs (miRNAs) and endogenous small interfering RNAs (endo-siRNAs), but is also critical for the degradation of toxic transposable elements. In this study, we investigated to which extent DICER1 is required for germ cell development and the progress of spermatogenesis in mice.

**Principal Findings:**

We show that the selective ablation of *Dicer1* at the early onset of male germ cell development leads to infertility, due to multiple cumulative defects at the meiotic and post-meiotic stages culminating with the absence of functional spermatozoa. Alterations were observed in the first spermatogenic wave and include delayed progression of spermatocytes to prophase I and increased apoptosis, resulting in a reduced number of round spermatids. The transition from round to mature spermatozoa was also severely affected, since the few spermatozoa formed in mutant animals were immobile and misshapen, exhibiting morphological defects of the head and flagellum. We also found evidence that the expression of transposable elements of the SINE family is up-regulated in *Dicer1*-depleted spermatocytes.

**Conclusions/Significance:**

Our findings indicate that DICER1 is dispensable for spermatogonial stem cell renewal and mitotic proliferation, but is required for germ cell differentiation through the meiotic and haploid phases of spermatogenesis.

## Introduction

Spermatogenesis is a complex biological process that involves the proliferation and differentiation of diploid spermatogonia into mature haploid spermatozoa. This process is divided into three sequential phases: first, the mitotic proliferation of spermatogonia gives rise to primary spermatocytes. These then undergo two meiotic divisions to generate haploid spermatids. The final phase, spermiogenesis, involves the morphological metamorphosis of early round spermatids into mature spermatozoa. As might be expected, the production of mature sperm requires a highly specialized control program of gene expression at both the transcriptional and post-transcriptional levels [1].

Small RNA molecules are important regulators of mRNA transcription, stability, turnover, processing, storage and translation (for review see [2]). These small RNAs can be classified into different categories based on their biogenesis, mechanism of action and function, and include microRNAs (miRNAs), endogenous small interfering RNAs (endo-siRNAs) and piwi-interacting RNAs (piRNAs). There is increasing evidence that small RNA-directed gene silencing pathways are essential for normal spermatogenesis [3], [4]. Not only have a large set of miRNAs [5], [6] and piRNAs [7], [8], [9] been identified in male germ cells, but all essential members of the RNA interference (RNAi) machinery, including DROSHA, DICER1, AGO2 (EIF2C2), PIWIL1(MIWI), PIWIL4 (MIWI2) and PIWIL2 (MILI) to name but a few, are expressed in meiotic and post-meiotic germ cells [10], [11].

DICER1 is a conserved RNAse III endonuclease that is essential for the processing of several classes of small RNAs, including miRNAs and endo-siRNAs, as well as for the degradation of toxic transposable elements [12], [13]. DICER1 has been found in nearly all organisms, and multiple studies have shown its essential role in animal development [14], [15], [16], [17], [18], [19], [20]. The functional relevance of DICER1 and miRNAs in spermatogenesis is only just beginning to be unravelled. Selective ablation of *Dicer1* in Sertoli cells leads to infertility, due to the complete absence of spermatozoa. More precisely, progressive testicular degeneration results from the defective maturation of Sertoli cells and their incapacity to properly support meiosis and spermiogenesis [21], [22]. However, the role of DICER1 in the male germ cell lineage in general, and specifically during spermatogenesis, is not as clear. Two groups have used Cre recombinase-expressing mice, TNAP-Cre (Tissue Non-Specific Alkaline Phosphatase) in an attempt to generate animals in which *Dicer1* is absent in germ cells [23], [24]. Hayashi and colleagues found that germ cell-specific deletion of *Dicer1* causes a defect in proliferation of male gonocytes and late adult infertility, likely due to spermatogenic arrest [23]. Maatouk and colleagues found that these mutant mice were subfertile, due to defects in both sperm motility and the transition from round to elongating spermatids [24]. Importantly, these results should be interpreted with caution as the TNAP-Cre transgenic mouse is neither fully penetrant (only ∼50% of germ cells express Cre), nor specific to germ cells. It should also be emphasized that the expression of TNAP-Cre begins as early as E10 [25], so that the primary effects appear in the primordial germ cell (PGC) population. It is therefore difficult to precisely interpret the mechanism and timing of any spermatogenesis defects occurring in the adult.

To investigate the precise roles of germ-cell DICER1 during spermatogenesis and to overcome the above-mentioned caveats of previous studies, we developed a mouse model in which the *Dicer1* gene was inactivated in a specific and fully penetrant manner in the male germ cell lineage. Our results indicate that *Dicer1* is required for normal spermatogenesis, since the deletion of *Dicer1* in male germ cells led to multiple defects in meiosis and spermiogenesis resulting in the absence of functional spermatozoa and complete infertility.

## Results

### Germ cell-specific deletion of *Dicer1* and miRNAs in *Ddx4-Cre;Dcr1^fx/fx^* testes

**Figure 1:**
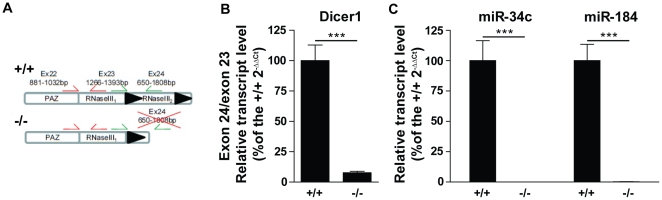
Germ cell-specific deletion of *Dicer1* and miRNA depletion in *Ddx4-Cre;Dcr1^fx/fx^* testes. (A) Diagram of exons 22 to 24 of the *Dcr1^fx/fx^* (+/+) and *Ddx4-Cre;Dcr1^fx/fx^* (−/−) alleles; the protein domains encoded by each exon are also noted. Exon 24 is flanked with loxP sites (black triangles), and excision occurs upon Ddx4-Cre recombinase expression. The primer pairs used in real-time RT-PCR for quantifying the excision of exon 24 (green) compared to exon 23 (red) are shown. (B) Histogram showing the exon 24 to exon 23 ratio of *Dicer1* mRNA in elutriated P60 spermatocytes isolated from +/+ (n = 3) and −/− (n = 3) testes; (C) Graph showing the expression of spermatocyte-specific miRNAs. Transcript abundance was quantified by real-time RT-PCR and normalized to 18s rRNA (B), U6 (C). Results are mean±SEM, ***p<0.0001 versus controls.

To investigate the functional relevance of DICER1 in spermatogenesis, we disrupted *Dicer1* in the male germ line by combining a conditional *Dicer1* allele (*Dcr1^fx^*) [15] with a *Ddx4*(MVH or Vasa) promoter-driven transgenic Cre line [26]. This *Ddx4-Cre* transgene has been reported to induce specific Cre-mediated recombination in >90% and >97% of spermatogonia by embryonic day (E)18 and postnatal day (P)3, respectively [26]. When *Ddx4-Cre*; *Dcr1^fx/wt^* males were mated with *Dcr1^fx/fx^* females, we observed that only ∼11% of the offspring were *Ddx4-Cre;Dcr1^fx/fx^* mutant males, instead of the expected ratio of 25% (for details see **Table S1**). This discrepancy from the Mendelian ratio is a consequence of early recombination events at the 1- or 2-cell stage, as previously described [26], leading to global embryonic Cre-mediated deletion of *Dicer1* and lethality around E7.5 [15], [27]. In the remaining viable *Ddx4-Cre;Dcr1^fx/fx^* males, *Dcr1* exon 24, which encodes most of the second RNAse III domain [15], was efficiently and specifically deleted in the germ cell lineage: using elutriated fractions containing spermatocytes isolated from P60 control and mutant *Ddx4-Cre;Dcr1^fx/fx^* mice, we found that levels of *Dcr1* transcripts that contained exon 24 were reduced by ∼93%; a proportion corresponding to the purity of the spermatocyte fractions (**Fig. 1A, B**). Furthermore, qRT-PCR showed complete loss of *miR-34c* and *miR-184*, two miRNAs expressed specifically in spermatocytes/spermatids ([28], and H. Kaessmann, personal communication; **Fig. 1C**). Together, these data show a complete loss of *Dicer1* and miRNA biogenesis in the male germ-cell lineage.

### Germ cell-specific deletion of *Dicer1* resulted in reduced testis size and sperm count, and complete male infertility

**Figure 2:**
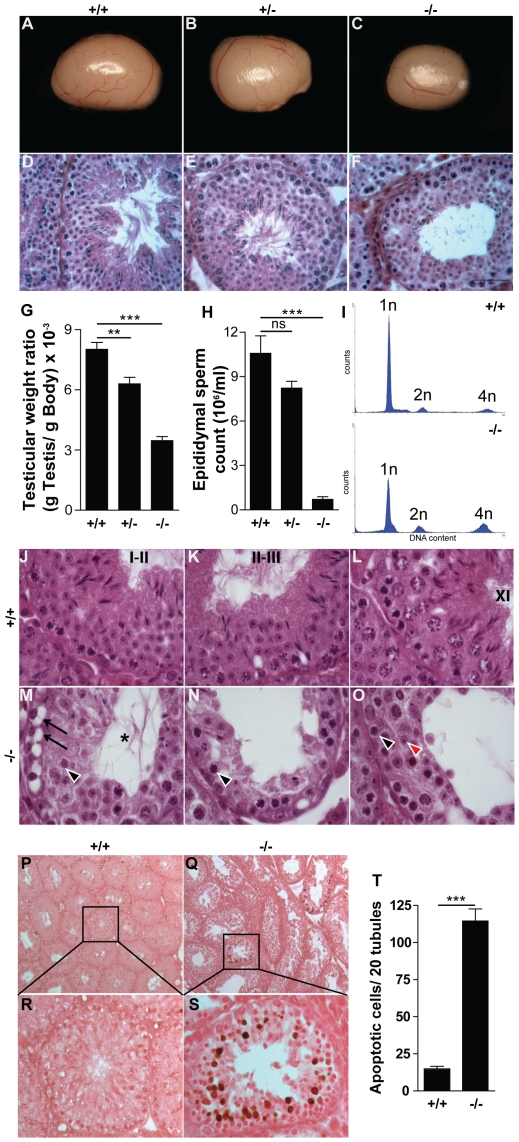
Reduction in testis size, apoptosis and near complete absence of mature spermatozoa in *Ddx4-Cre;Dcr1^fx/fx^* (−/−) testes. At P60, testes from −/− (n = 16) mice (C) showed a 55% reduction (G) in weight compared to control *Dcr1^fx/fx^* (+/+; n = 11) (A) and *Ddx4-Cre;Dcr1^fx/wt^* (+/−; n = 6) (B) littermates. H&E staining of testes sections (D–F) revealed near complete absence of mature spermatozoa and elongated spermatids in −/− testes. (H) Epididymal sperm count analysis showed a ∼99% decreased in −/− epididymides. (I) DNA content histogram of P60 +/+ (upper panel) and −/− (lower panel) testes sorted by FACS using propidium iodide. (J–L) H&E staining of +/+ testes showing stage I–II (J), II–III (K) and XI (L) seminiferous tubules. (M–O) −/− tubules are devoid of elongated spermatids and show numerous seminiferous epithelium defects such as vacuolization (arrows), and Sertoli cell cytoplasmic lumen extensions (asterisk). Sparse (2–3) round spermatids (arrowhead in M), and zygotene spermatocytes (arrowhead in N) were found in tubules. (O) Germ cell association is disturbed in tubules as shown by the presence of leptotene spermatocyte (black arrowhead) and (2–3) few round spermatids (red arrowhead). Global TUNEL/eosin staining of P60 +/+ (P) and −/− (Q) testes revealed massive apoptosis (brown nuclei) in some specific −/− seminiferous tubules. Histological analysis indicated that meiotic cells are the most abundant apoptotic population found in −/− (S). (T) The overall number of TUNEL-positive cells is increased ∼8-fold in −/− compared to +/+ individuals. Results are mean ±SEM, *p<0.05, **p<0.01, ***p<0.001 versus controls. ns: not significant. Scale bar: 50 µm (A–F).

*Ddx4-Cre;Dcr1^fx/fx^* males were viable and grew normally displaying normal external genitalia, when compared to control littermates. At P60, testes in which *Dicer1* was depleted in germ cells showed a reduction in size (compare **Fig. 2A** and **B** with **C**) and a 55% decrease in weight compared to control *Ddx4-Cre;Dcr1^fx/wt^* and *Dcr1^fx/fx^* littermates (107±7 mg versus 238±9 mg and 196±11 mg respectively, for details see **Table S2** and **Fig. 2G**). Internal reproductive organs (*i.e.* seminal vesicles) were normally developed and, importantly, plasma testosterone levels were unchanged between experimental and control groups (**Table S2**). While control *Ddx4-Cre;Dcr1^fx/wt^* and *Dcr1^fx/fx^* males were fertile, *Ddx4-Cre;Dcr1^fx/fx^* mutant males were found to be sterile, although they were sexually active and produced vaginal plugs in female partners (data not shown). Histological analysis and sperm count in mutant individuals revealed that sperm production was drastically altered in the mutant testis. Indeed, mature spermatozoa were rarely found in the tubule's lumen due to a near complete absence of elongated spermatids (**Fig. 2F**). As a consequence, epididymal ducts were almost completely devoid of mature spermatozoa, whereas exfoliated germ cells were found in the lumen of mutant epididymides (data not shown). Finally, epididymal sperm count analysis revealed a ∼99% decrease in the number of spermatozoa in *Ddx4-Cre;Dcr1^fx/fx^* males compared to control *Ddx4-Cre;Dcr1^fx/wt^* and *Dcr1^fx/fx^* littermates (0.141±0.041 *10^6^/ml versus 8.39±0.5 *10^6^/ml and 10.58±1.175 *10^6^/ml respectively; **Fig. 2H** and **Table S2**).

### *Dicer1* mutant mice exhibit abnormal seminiferous tubular cell association, germ cell apoptosis and organization defects

Adult seminiferous tubules contain several types of germ cells; each cell type is layered within the tubule in a centripetal manner and germ cells are arranged in typical cellular associations within the seminiferous tubules known as stages of the seminiferous epithelium cycle. In mice, a spermatogenetic cellular association is divided into 12 stages (from I to XII; [29]). An in depth analysis of the mutant seminiferous epithelium histology and cytology revealed multiple defects. First, the relative proportions of the different populations of germ cells are clearly unbalanced (**Fig. 2M, N** and **O**). Using propidium iodide incorporation followed by flow cytometry analysis, we found that the proportion of haploid (1n) cells was reduced by ∼30%, while tetraploid (4n) cells showed a 2.5-fold increase in *Ddx4-Cre;Dcr1^fx/fx^* males (**Fig. 2I**). This result corroborates our analysis of the mutant testis histology, in which almost no elongated spermatids were found (**Fig. 2N**). Secondly, vacuoles (**Fig. 2M**, arrows) were numerous in the mutant epithelium, likely due to germ cell sloughing, as were the Sertoli cell cytoplasmic extensions in the lumen (**Fig. 2M**, asterisk). As a possible consequence of these multiple cellular defects, we observed a reduction in the small diameter of mutant seminiferous tubules when compared to *Dcr1^fx/fx^* littermates (123±2 µm versus 167±8 µm, **Table S2**). Another important characteristic of mutant seminiferous tubules was the disturbance of the synchronization of germ cell associations (**Fig. 2O**). For instance, we observed leptotene spermatocytes (**Fig. 2O**, black arrowheads), which are ordinarily found in stage IX–X tubules, associated with round spermatids (**Fig. 2O**, red arrowhead), normally found in stage I–VIII tubules. These observations prevented the clear identification of specific tubule stages in *Dicer1* mutant testes. Interestingly, although global germ cell organization was completely disturbed, Sertoli cell nuclear organization and number appeared normal (**Fig. S1**; **Table S1**).

**Figure 3:**
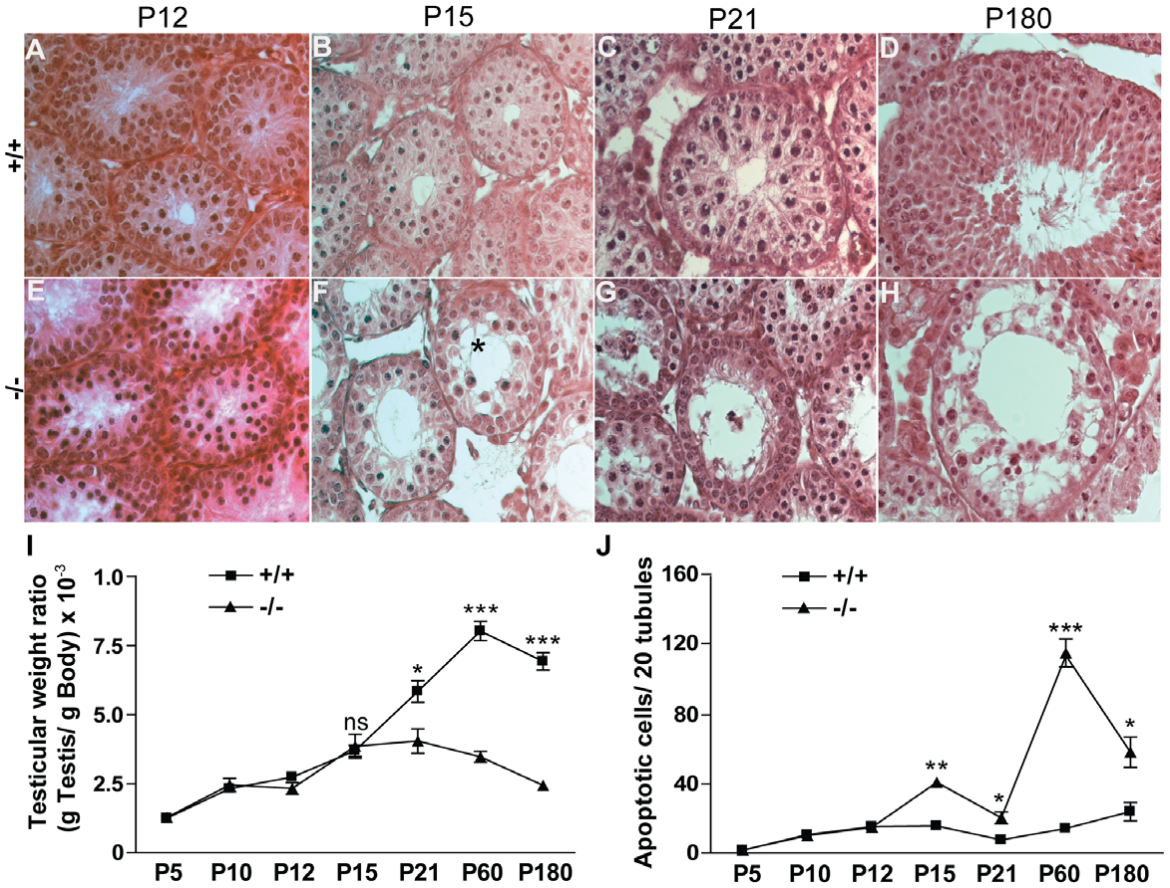
Tubular defects appear as early as P15 in *Ddx4-Cre;Dcr1^fx/fx^* mutant testes. H&E staining of control (+/+) (A, B, C, D) and mutant (−/−) (E, F, G, H) testes at P12 (A&E), P15 (B&F) ,P21 (C&G) and P180 (D&H). The first anatomical defects including vacuolization (asterisk), apoptosis and germ cell disorganization appeared at P15 (F) and worsened by P21 (G). In aging individuals, at P180 (D&H), tubular histology is strongly affected, with numerous vacuoles and few germ cells remaining. Reduced testis weight ratio (I) correlated with an increase in apoptotic rate (J) during the first spermatogenic wave. For I and J, numbers of animals were n = 5 minimum for each genotype. Results are mean ±SEM, *p<0.05, **p<0.01, ***p<0.001 versus controls. ns: not significant.

The significant reduction of post-meiotic germ cells (**Fig. 2I**) in mutant seminiferous tubules suggested that germ cell survival may be affected in the absence of DICER1. A TUNEL assay revealed that apoptosis was 8-fold higher in mutant testes compared to control seminiferous tubules (115±8 apoptotic cells/20 tubules versus 15±1 apoptotic cells/20 tubules and **Fig. 2T**). Interestingly, apoptotic cells were not distributed evenly but usually localized in specific seminiferous tubules (**Fig. 2Q and S**). Histological analyses involving double immunostaining for TUNEL and a subset of germ cell markers (γH2AX (phosphorylated H2AX), H3K9me3 (tri-methylated Lysine9 Histone 3), alpha tubulin or pH 3 (phosphorylated-Ser10 Histone 3)), suggested that apoptotic cells mainly comprise pachytene spermatocytes (**Fig. S2** and data not shown). Furthermore, aging mutant testes, at P180, showed high apoptotic cell numbers with extensive tubular defects, although Sertoli Cell Only (SCO) tubules were not observed (**Fig. 3D and H**). In short, our data indicate that depletion of DICER1 RNAse activity in the germinal compartment of the testis leads to significant defects in spermatogenesis, affecting both the survival and differentiation of male post-mitotic germ cells.

### *Ddx4-Cre;Dcr1^fx/fx^* males show meiotic defects during spermatogenesis

To further characterize the events that lead to infertility in *Ddx4-Cre;Dcr1^fx/fx^* mutant mice, we compared the development of control and mutant testes during the early phases of the first spermatogenic wave, from P5 to P21. No discernible abnormality was found in mutant testes during the mitotic phase of spermatogonia A (P5), when spermatogonia B enter meiosis I (P10) (**Fig. S3**), or when spermatocytes are in the zygotene/pachytene transition (P12) (**Fig. 3A and E**). The first histological abnormalities began to appear at P15 with the presence of germ cell sloughing and Sertoli cell cytoplasmic extensions (**Fig. 3B and F**). At this stage, apoptosis also increased by ∼2.5-fold in mutant testes in comparison with control individuals (41±1 apoptotic cells/20 tubules versus 16±3 apoptotic cells/20 tubules respectively, **Fig. 3J**). By P21, in wild-type testis, meiosis I is complete and round spermatids begin to differentiate, which was not the case is in mutant testis. A difference in testis weight also became apparent (23.75±3.1 mg versus 51.11±6 mg respectively, **Fig. 3I**), which reflects seminiferous tubule deterioration and increased apoptosis (21±3 apoptotic cells/20tubules versus 8±2 apoptotic cells/20 tubules, **Fig. 3J**).

**Figure 4:**
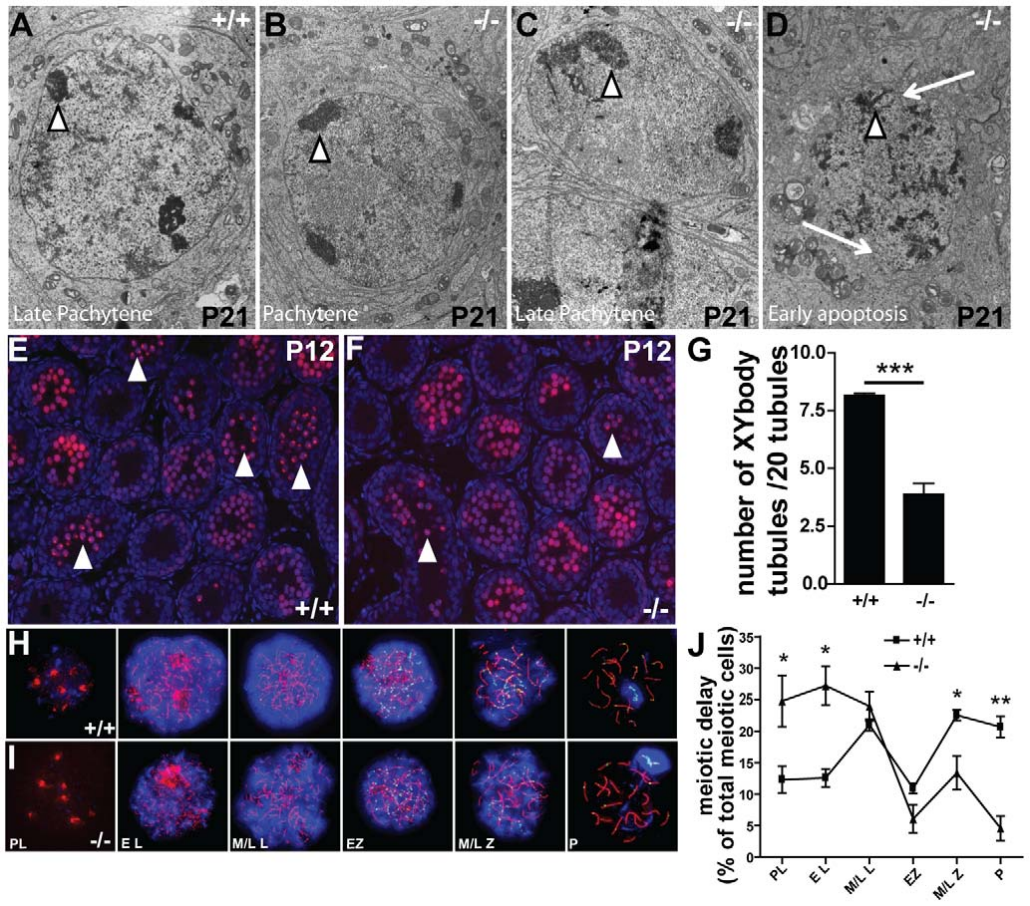
Meiotic progression defects in *Ddx4-Cre;Dcr1^fx/fx^* (−/−) mutant testes. Representative transmission electron micrographs from P21 control (A) and mutant (B–D) testes. Note the enlarged perinuclear heterochromatin areas (arrowheads) and the irregular and abnormal nucleus shape (arrows) in mutant pachytene spermatocytes typical of apoptotic cells. (E, F) Anti-γH2AX staining (red), present in the whole nucleus in early meiotic stages and restricted to the XY body from pachytene phase, revealed a reduction in the number of tubules containing XY body positive cells (arrowheads, punctual red staining) within −/− testis (F) compared to wild-type (E). DAPI (blue) was used for nuclear staining. (G) shows the quantification of XY body positive tubules per 20 tubules. Anti-SYCP3 (red), DMC1 (green) and γH2AX (blue) staining of chromosomal spread preparations from control (H), and mutant (I) P12 testes was used to quantify meiotic prophase I cells. (J) Note the higher number of early meiotic cells (i.e., Preleptotene and leptotene) and the reduced number of late meiotic cells (i.e. mid/Late zygotene and pachytene) in −/− cell preparations suggesting a delayed progression of germ cells into meiosis. Results are mean ±SEM, ns = not significant, *p<0.05, **p<0.01, ***p<0.001 versus controls. PL: Pre-leptotene, EL: Early leptotene, M/L L: mid/late leptotene, EZ: early zygotene, M/L Z: mid/late zygotene, P: Pachytene.

In order to better characterize the apoptotic cell population present within the developing tubules, we performed electronic microscopy (EM) of control and mutant testes at the end of prophase I (P21; **Fig. 4A, B, C, D**). In *Dicer1*-depleted germ cell individuals, most of the pachytene spermatocytes showed apoptotic features (e.g. misshapen nuclear membrane and fragmented heterochromatin, arrows and arrowheads in **Fig. 4D**, respectively), and those that survived prophase I rarely reached the metaphase step. In addition, metaphase plates appeared abnormal (data not shown). Zygotene (not shown) and pachytene spermatocyte nuclei in *Ddx4-Cre;Dcr1^fx/fx^* mutant tubules seemed to contain abnormally large perinuclear heterochromatin areas (arrowheads in **Fig. 4B and C**). We used ser-10 phosphorylation of histone 3 as a specific marker of cells in metaphase (*i.e.* spermatogonia and late meiotic cells; [11]) to compare mutant individuals to control littermates at P60. This revealed an increased number of cells in meiotic metaphase in the tubules of the mutant group (**Fig. S4**). These findings indicate that spermatocytes may be partially blocked in the late prophase I phase in *Ddx4-Cre; Dcr1^fx/fx^* mice.

Since pachytene spermatocytes seemed to be affected by the deletion of *Dicer1*, we analysed the expression of γH2AX at P12, during the zygotene to pachytene transition. During the normal zygotene phase, γH2AX localizes to the whole nucleus, but in the subsequent pachytene stage remains only in the XY body (or sex body) where X and Y-linked genes undergo transcriptional silencing [30]. At this stage, we found that the number of pachytene spermatocyte-containing tubules was reduced by ∼2-fold (3.88±0.4 versus 8.16±0.1 XY body-positive tubules/20 tubules respectively; **Fig. 4G**) in mutants compared to control littermates (arrowheads in **Fig. 4E and F**). Chromosomal spread preparations from germ cells collected at P12 confirmed the reduced number of pachytene spermatocytes in mutant testes (**Fig. 4 H, I, J**), whereas, pre-leptotene and leptotene cells were more numerous compared to controls. No difference in the meiotic process was observed, such as synapsis (SYCP3 staining) or repair (DMC1 punctuate staining). Taken together, these data show that the loss of *Dicer1* in germ cells severely impairs the first spermatogenic wave, by delaying the transition from the leptotene to the zygotene/pachytene stages of the first meiotic prophase, and by increasing apoptosis in mid/late pachytene stage spermatocytes.

### Spermiogenesis is impaired in *Dicer1* mutant mice

**Figure 5:**
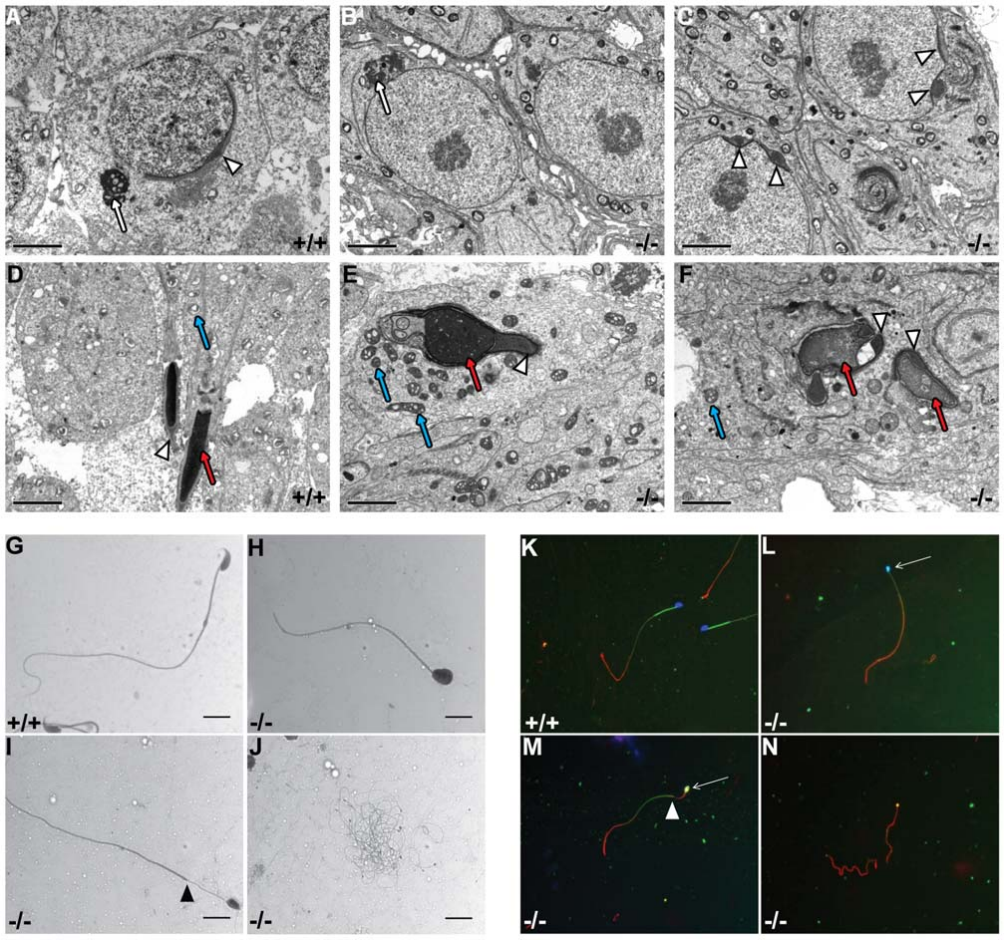
Spermiogenic and sperm alterations in *Ddx4-Cre;Dcr1^fx/fx^* (−/−) testes. Representative transmission electron micrographs from P60 control (A&D) and mutant (B, C, E & F). In round spermatids (A–C), chromatoid body is normally shaped and located near the nucleus (red arrows), whereas acrosome is fragmented in mutant (arrowheads). In elongated spermatids (D–F), nuclear shape (red arrows), chromatin condensation, and acrosome (arrowheads) are abnormal in −/− (E and F) mice. Mitochondria showed hyperplasia of the intermembrane space or cristae (blue arrows, E). Scale bar: 2 µm. H&E staining of epididymal sperm spreads of +/+ (G) and −/− (H, I and J) adult mice. In contrast to +/+ animals (G), spermatozoa of −/− mice exhibited multiple defects such as head morphology, mid-piece defects (H and I) and reduced overall size (J). Black arrowhead shows the ectopic localization of the mid-piece in (I). Scale bars: 10 µm. Immunostaining of epididymal sperm spreads of control +/+ (K) and −/− (L, M and N) mice using DAPI staining to label nuclear DNA (blue), Mitotracker as a mitochondrial marker (green) and anti-α-tubulin to label the flagellum (red). Spermatozoa of −/− mice exhibited head morphology defects (L, M and N). White arrows show that some mutant spermatozoa display abnormal shaped nuclei and co-localized ectopic mitochondrial staining. White arrowhead in (M) indicates the ectopic localization of the mid-piece mitochondria compared to control individuals (K). Images were taken with a 40× objective.

To characterize the morphological defects in the developing germ cells after meiosis, we performed an EM study on testis sections at P60. The few spermatids found in *Ddx4-Cre;Dcr1^fx/fx^* mutant mice displayed various defects; the acrosome of round spermatids was fragmented in *Ddx4-Cre;Dcr1^fx/fx^* with multiple acrosomal granules tethering the nucleus (**Fig. 5C**, arrowheads) instead of a single granule found in control testes (**Fig. 5 A**, arrowhead). Interestingly, we found no apparent defects in the overall morphology and position of the chromatoid body, a cloud-like structure thought to be the site of RNA processing and/or storage (white arrows in **Fig. 5A** and **B**). The few remaining elongated spermatids of mutant animals showed abnormal head shape and chromatin condensation (red arrows in **Fig. 5E** and **F**). Acrosomes were also misshapen and fragmented (compare arrowheads in **Fig. 5 E and F** with **D**). Finally, mitochondria displayed abnormal shape and dilated cristae or intermembrane space, which appeared as a dense structure, compared to control spermatids (blue arrows, **Fig. 5 D, E, F**).

Since round and elongated spermatids showed significant defects in acrosome formation and nuclear condensation we analysed further the morphology of the remaining epididymal sperm (**Fig. 2 H**). All of the few spermatozoon-like structures found in the epididymides of P60 mutant individuals displayed drastic morphological defects of the head and flagellum (**Fig. 5G, H, I, J**). Using a dye specifically staining mitochondria, we evidenced a heterogeneous and aberrant localisation of these organelles in the mutant spermatozoa, with mitochondria surrounding the nucleus (arrows in **Fig. 5L and M**) or mislocalized within the flagella (arrowheads in **Fig. 5M**). In addition to the misshapen but normally-sized spermatozoa present in the mutant sperm, we were able to see what resembled small pin-head spermatozoa (**Fig. 5N**; α-tubulin red staining). Nevertheless, these structures were devoid of nuclei and harbour a faint and sparse mitochondrial staining. None of the mutant spermatozoa were motile when examined by visual inspection with a light microscope (data not shown). Our results indicate that functional DICER1 is required for normal spermiogenesis, since its ablation in germ cells leads to a reduced number of haploid germ cells and extensive defects in global sperm morphology.

### Expression of transposable elements increases in spermatocytes lacking DICER1 activity

**Figure 6:**
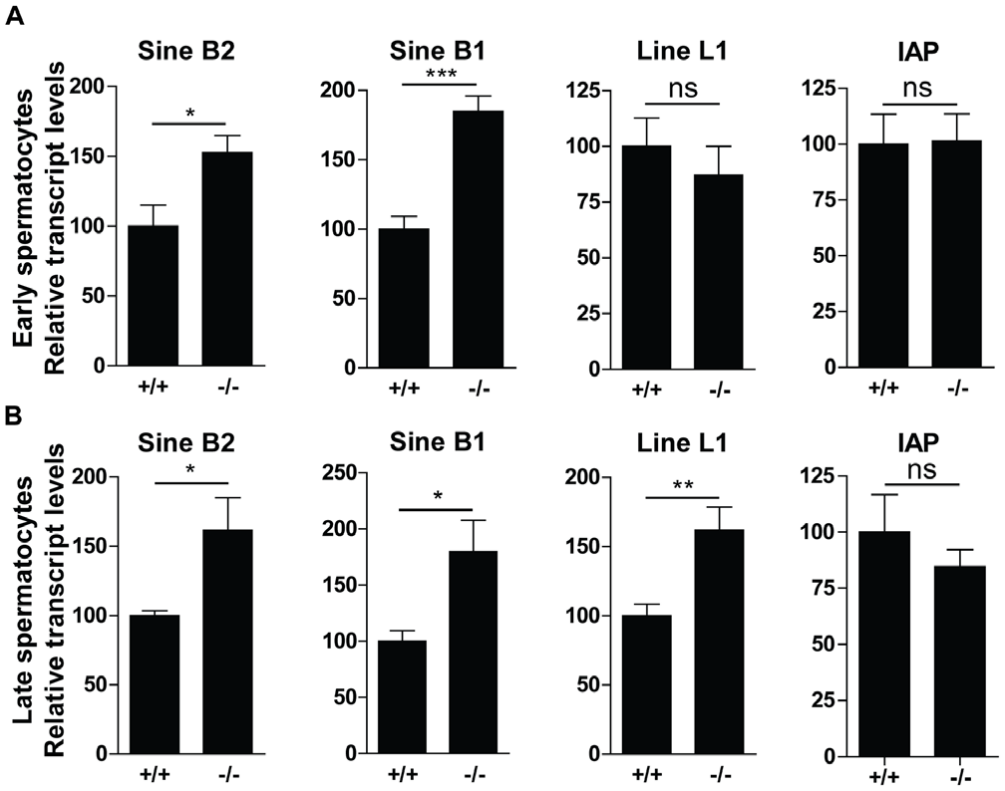
Deregulation of transposable elements in *Ddx4-Cre;Dcr1^fx/fx^* (−/−) mutant spermatocytes. Quantitative real-time PCR performed on elutriated germ cell fractions enriched either in leptotene/zygotene/early pachytene spermatocytes (A) or late pachytene/diplotene spermatocytes (B) originating from +/+ and −/− testes at P60. Results are mean ±SEM, ns = not significant, *p<0.05, **p<0.01, ***p<0.001 versus controls.

In addition to its essential role in miRNA biogenesis, DICER1 has been implicated in the control of repetitive elements. The recent discovery that DICER1 is critical for cell survival by degrading toxic retrotransposon transcripts in retinal pigment epithelium [12] raised the possibility that DICER1 might fulfil the same function in the germ cell lineage. To investigate this possibility, we examined the expression levels of IAP (intracisternal A particle element), LINE1 (long interspersed nuclear element 1) and SINE B1 and B2 (short interspersed nuclear element B1 and B2) by quantitative RT-PCR in two groups of P60 elutriated germ cells (**Fig. 6**). While transcript levels for transposable elements LINE1 and IAP were unaffected, we observed a significant up-regulation of SINE B1 and SINE B2 transcripts in enriched populations of leptotene/zygotene/mid-pachytene spermatocytes (1.8-fold increase for SINE B1 and 1.5- fold for SINE B2) and late-pachytene/diplotene spermatocytes (1.8-fold increase for SINE B1 and 1.6- fold for SINE B2) prepared from mutant testes. Whether the ∼1.5–2 fold increase in SINE transcripts affect germ cell survival and/or spermatogenesis remains an open question.

## Discussion

DICER1 is essential for the processing of several classes of small non-coding RNAs, including miRNAs and endo-siRNAs, and for the degradation of retrotransposon transcripts. In this study we used the Cre/Lox system to conditionally inactivate *Dicer1* in male germ cells. We found that DICER1 is not required for spermatogonia stem cell renewal and mitotic proliferation, but is essential for the meiotic and haploid phases of spermatogenesis. The first defects appeared as early as P12 when we observed a delayed progression of prophase I, followed at P15 by an increase in apoptosis of mid/late pachytene stage spermatocytes. Not all spermatocytes died by apoptosis, as a small fraction of these completed meiosis. The transition from round spermatids to mature spermatozoa was also severely affected since the few spermatozoa that did form in mutant animals (at 1% of control levels) were immobile and misshapen, exhibiting irreversible morphological defects due to disturbance in acrosome formation, nuclear condensation in the few remaining round and elongated spermatids.

The loss of *Dicer1* in male germ cells thus led to a complex and severe phenotype, which likely represents the sum of low impact defects that accumulate at various meiotic and post-meiotic stages. This type of phenotype, resulting from the cumulative impairment of several developmental cellular processes and increased cell death, has in fact been observed in numerous conditional *Dicer1* knockouts [15], [16], [17], [31]. Conditional removal of *Dicer1* in the male germ cell lineage led to various phenotypes whose severity depends on the efficiency of Cre recombination and/or the stage at which DICER1 has been inactivated. Initial studies using a *Tnap-Cre* transgene has been problematic due to early inactivation (E10) as well as the partial (50%) and non-specific Cre-mediated expression [25]. As a result of inefficient Cre recombination, *Tnap-Cre;Dicer^fx/fx^* mice were found either fertile or subfertile and it was a rather difficult task to evaluate precisely the impact of DICER1 on sperm production [23], [24]. The use of a specific, fully penetrant Cre line such as the *Ddx4-Cre* transgene was instrumental to investigate in depth the function of DICER1 in spermatogenesis. Interestingly, ablation of DICER1 in germ cells at different development stages using two different spermatogonia-specific Cre mice (the *Ddx4-Cre* and *Ngn3-Cre*) revealed a gradation in severity of the reproductive phenotype. *Ddx4-Cre* and *Ngn3-Cre* transgenes are both efficient at deleting DICER1 in gonocytes at ∼P0 and in spermatogonia at ∼P5–P7, respectively (Korhonenet al, co-submitted manuscript). However, germ cell-specific removal of DICER1 using a *Ngn3-Cre* transgenic resulted in a phenotype slightly milder than the *Ddx4-Cre;Dicer1^fx/fx^* described here. Both mutant males were infertile and exhibited similar defects in spermato- and spermiogenesis but the meiotic phenotype was more severe and precocious in *Ddx4-Cre;Dicer1^fx/fx^* animals with defects in prophase I progression and a lower number of remaining spermatozoa. Since small RNAs are stable molecules, we hypothesize that ablation of DICER1 in gonocytes at ∼P0 will deplete the pool of the remaining miRNAs or endo-siRNAs more extensively than in P5–P7 spermatogonia thus affecting spermatogenesis and sperm production more severely.

There is no doubt that DICER1 is essential for spermatogenesis and sperm production. However, emerging from these findings is the essential question of which DICER1 activity is crucial for germ cell function. In other words, the relative contribution of miRNAs versus other DICER1-dependent non-coding RNAs to the phenotype remains unclear. DICER1 is essential for both endo-siRNA and canonical miRNA biogenesis, but not for piRNA production (for a review see [2]). Recent expression profiling analyses using RNA sequencing technology revealed that male spermatogenic cells expressed numerous classes of small non-coding RNAs including miRNAs and endo-siRNAs [5], [6]. Whether the infertility phenotype observed in our *Ddx4-Cre;Dcr1^fx/fx^* is consecutive either to the loss of endo-siRNAs and/or miRNAs is still uncertain. While revising this manuscript, it has just been reported that germ cell inactivation of *Dicer1* or *Drosha*, an RNase III enzyme required for canonical miRNA biogenesis but not for endo-siRNA processing, both resulted in male infertility due to oligozoopsermia or azoospermia caused by constant depletion of phachytene spermatocytes and spermatids [6]. The similar phenotype observed in *Drosha* knockout testes when compared with the *Dicer* mutants suggests a role for miRNAs in regulating spermatogenesis. These results are different to those found in female germ cells, where both miRNAs and endo-siRNAs are also present in the developing oocytes [32], [33]. While mouse oocytes lacking *Dicer1* arrest in meiosis I due to disorganized spindles and defects in chromosomal alignment [34], [35], oocytes deficient for *Dgcr8*, an RNA-binding protein that assists DROSHA in the processing of microRNAs, mature normally and do not impair early embryonic development [36]. This striking difference in phenotype between *Dicer1*- and *Drosha*-deficient oocytes suggests that endo-siRNAs, rather than miRNAs, are essential for oocyte maturation and the early stages of mammalian development [6]. Another important difference between male and female germ cells is the timing of defects. In oocytes lacking *Dicer1* a specific defect is observed at metaphase, affecting either the spindle assembly checkpoint (SAC) and/or the anaphase-promoting complex (APC/C). Indeed, it was demonstrated that DICER1 is not required for female germ cell development but only for meiotic maturation [37]. Our results clearly show that unlike oocytes, defects in spermatogenesis appear earlier during prophase I.

The chromatoid body (CB) is a dynamic, cloud-like, dense structure located in the cytoplasm of round spermatids, and is thought to be the site of RNA processing and/or storage [38]. This cytoplasmic perinuclear organelle contains various types of RNAs, including piRNAs, mRNAs and miRNAs as well as various RNA-binding proteins or proteins involved in RNA processing pathway (e.g. DDX4, PIWIL1, PIWIL2, DICER1, tudor domain proteins such as TDRD6 and TDRD7; [39]). The potential role of the CB in the mRNA regulation [38], together with the multiple cumulative meiotic and post-meiotic defects found in *Dicer1*-depleted germ cells, suggested the hypothesis that the CB structure and/or its function may be affected in *Dicer1*-depleted germ cells. EM studies of testes at P60 revealed that the CB was present, and normally shaped in round spermatids lacking *Dicer1*, this suggests that DICER1 is not essential for the formation of the CB. Whether its molecular composition and/or its function in RNA regulation is affected in mutant germ cells still remains unclear.

The mouse and human genomes are crowded with transposable elements that account for 30 to 40% of their total size [40]. Different silencing mechanisms have been developed in germ cells to suppress their activities, including DNA methylation [41] and RNAi-triggered silencing (for a review see [42]). The large set of piRNAs expressed in spermatocytes highlights the importance of these small RNAs in protecting male germ cells from transposable elements [43]. While piRNA biogenesis requires neither DROSHA nor DICER1, there is substantial evidence that regulation of transposable element expression includes DICER1-dependent mechanisms [44], [45], [46]. Several retrotransposons from the MT and SINE families were shown to be significantly up-regulated in mouse oocytes lacking *Dicer1* [34]. Recently, DICER1 was demonstrated to be involved in retrotransposon transcript degradation. *Dicer1* ablation in retinal pigmented epithelium (RPE) induced accumulation of alu/SINE RNAs in both human and mouse cells, resulting in cytotoxicity and RPE degeneration [12]. Our genetic analysis suggests that the SINE family of retrotransposons is up-regulated in spermatocytes lacking *Dicer1*. Whether this 1.5- to 2-fold increase in SINE expression is sufficient to affect survival and/or specific stages of spermatogenesis remains unclear and requires further analysis. In this perspective, high throughput RNA sequencing combined with an in-depth differential proteomic analysis of *Dicer1*- and *Dgcr8*-depleted germ cells would be instrumental for a better comprehension of the roles and impact of small non-coding RNAs in the production of mature sperm.

## Materials and Methods

### Animals

*Dcr1^flox^ (Dcr1^fx^)* and *Ddx4:Cre (Mvh-Cre)* mice were kindly provided by B.D. Harfe and D.H. Castrillon respectively, and were genotyped as described [15], [26]. To achieve selective inactivation of *Dicer1* in germ cells, we mated transgenic male mice expressing Cre recombinase under the control of the *Ddx4* promoter with female mice carrying two floxed *Dicer1* alleles in order to generate 50% *Ddx4-Cre;Dcr1^fx/wt^* and 50% *Dcr1^fx/wt^* mice. *Ddx4-Cre;Dcr1^fx/wt^* males were mated with *Dcr1^fx/fx^* females in order to produce *Ddx4-Cre;Dcr1^fx/fx^* as well as *Dcr1^fx/fx^* and *Ddx4-Cre;Dcr1^fx/wt^* control littermates. The genetic background of these mice is mixed C57Bl/6J and SV129. Protocols for the use of animals were approved by the commission d'Ethique de l'Expérimentation Animale of the University of Geneva Medical School and the Geneva Veterinarian Office.

### Isolation of testicular cells

Male germ cells were obtained from P60 adult mice (n = 3, each pool of germ cells was isolated from either four pairs of *Ddx4-Cre;Dcr1^fx/fx^* mutant testes or two pairs of *Dcr1^fx/fx^* control testes) by mechanical disruption and liberase treatment (Roche Applied Science). Cells were elutriated and separated into multiple fractions according to [47] and subsequently frozen for further analysis.

### RNA extraction and Quantitative Real-Time PCR

Total RNAs from elutriated germ cells were extracted using Trizol (Sigma-Aldrich) according to the manufacturer's instructions. For further quantification of repetitive elements associated RNAs, extracted RNA was subjected to DNAse treatment using the Turbofree DNA kit (Ambion). Quantification of transcript levels was performed using the Kapa Sybr Fast kit (Kapa Biosystems) with primers for *Dicer1* already described in [48], SINE B1, B2 and LINE L1 RNA primers described in [49] and IAP primers sequences came from [50]. Taqman miRNA kit (Applied Biosystems) was used for quantification of miRNAs according to the manufacturer's instructions using primers for miR-34c and miR-184 (for probe sequences see Applied Biosystems website). Data were analyzed using the 2^−ΔΔCt^ method as described in [51]; normalization was performed with 18s rRNA levels for repetitive elements and mRNA transcripts, whereas U6 small RNA was used for miRNA level normalization. Each assay was performed at least in three independent technical and biological replicates.

### Measurement of hormonal plasma levels

Blood was collected by cardiac puncture from P60 mice. Plasma samples were stored at-20°C and used subsequently to assess the levels of LH, FSH and testosterone. Hormone levels were measured in individual adult plasma samples (n = 3–9) from P60 adult mice. LH was measured by RIA, using a commercially available kit supplied by IDS (LH RIA CT # AHR002, Liège, Belgium), whereas FSH was measured by IRMA, using a kit from the same supplier (FSH IRMA CT # AHR004). Testosterone concentrations were assayed using a kit from MP Biomedicals (Testo DA kit, CT number 07-189102, Eschwege, Germany). Intra- and inter-assay coefficients of variation (CVs) of all three assays were less than 5% and 10%, respectively.

### Sperm analysis and Immunofluorescence assays

Epididymal sperm count was performed with sperm extracted from the caudal epididymis and ductus deferens of adult (P60) male mice and was analyzed for its concentration as previously described [52]. Ploidy was assessed on *Ddx4-Cre;Dcr1^fx/fx^* and *Dcr1^fx/fx^* P60 testis using Propidium Iodide incorporation as described in [53]. Epididymal sperm from *Ddx4-Cre;Dcr1^fx/fx^* and *Dcr1^fx/fx^* was spread on SuperFrost Plus glass slides, fixed 10 minutes at room temperature (RT) with 4% paraformaldehyde (PFA), then permeabilized in PBS/triton-X100 0.5% and incubated for 1 hour at RT with anti-alpha-Tubulin (Abcam clone B-5-1-2, 1∶500). For fluorescent staining an Alexa594 secondary antibody (Invitrogen) was used for signal revelation; nuclei were counterstained using DAPI and Mitotracker (1 µM, Invitrogen) was used for mitochondrial staining.

### Histology and Immunochemistry

Tissues were fixed overnight either in 4% PFA or in Bouin's fixative and embedded in paraffin. Five-µm sections were stained with haematoxylin and eosin (H&E) or processed for immunohistochemistry (IHC). For IHC analysis, PFA-fixed sections were incubated overnight at 4°C with the following antibodies: anti-GATA4 (Santa Cruz Biotechnology, sc-9053, 1∶500), anti-pH 3(Ser10) (Millipore, Cat#06-570, 1∶500), anti-γH2AX (Calbiochem, Cat#dr-1017, 1∶500) and anti-H3K9me3 (Millipore, Cat#07-523, 1∶500). For fluorescent staining, Alexa-conjugated secondary antibodies (Invitrogen) were used for signal revelation and sections were counterstained using DAPI. All images were obtained either with a Zeiss Axioscope microscope and processed using the AxioVision LE software.

### Apoptosis Assays

Apoptotic assays were performed by TdTmediated X-dUTP nicked labeling (TUNEL) reaction using the Apoptag kit (Millipore) stained with either DAB chromogen and counterstained with eosin, or with Permanent Red or anti-Digoxygenin coupled to fluorescein and counterstained with DAPI. The percentage of apoptotic, TUNEL positive cells within seminiferous tubules was expressed as the average number of apoptotic cells within 20 seminiferous tubes. A minimum of 100 seminiferous tubules were counted per testis (5 sections/testis) and at least 3 animals per genotype per age were assessed.

### Chromosome Spread Preparation and Immunostaining

Spermatocytes nuclear spreads were prepared and stained as previously described [54]. The primary antibodies used were: guinea pig anti-SYCP3 (gift from C. Heyting, 1∶25 000), mouse anti-γH2AX (Upstate, 05-636, 1∶25 000) and rabbit anti-DMC1 (Santa Cruz Biotechnology, H100, 1∶200). Secondary antibodies were AlexaFluor-488 and AlexaFluor594 conjugates (Molecular Probes).

Digital images were obtained by using a cooled CCD camera, Coolsnap HQ (Photometrics), coupled to a Leica DMRA2 microscope using the same exposure time for all acquisitions. Each colour signal was acquired as a black-and-white image using appropriate filter sets and was merged with Photoshop Imaging software.

### Electron microscopy

P60 and P21 testes from *Ddx4-Cre;Dcr1^fx/fx^* and *Dcr1^fx/fx^* individuals were fixed in 0.1 M Na Cacodylate with 4% Glutaraldehyde in PBS. After fixation with 1% osmium tetroxide, the testes were embedded in epoxy resin (Glycidether 100, Merck). Selected areas were sectioned, stained with 5% uranyl acetate and 5% lead citrate, and visualized on a JEOL 1200 EX transmission electron microscope.

## Supporting Information

Figure S1 **Sertoli cells organization and number are not affected in testes lacking** ***Dicer1*** **in the germinal compartment.** Gata-4 immunostaining (red) revealed the presence of Sertoli cells nuclei at the edge of tubules in *Dcr1^fx/fx^* (+/+) control individuals (A) as well as in *Ddx4-Cre;Dcr1^fx/fx^* mutant mice (−/−) (B), indicating that Sertoli cells organization is not affected by Dicer1 depletion in germ cells. (C) The number of Sertoli cells within seminiferous tubules is also unaffected (n = 3 animals per genotype; a minimum of 20 tubules were analyzed per individual).
(TIF)

Figure S2 **Apoptotic cell population display pachytene spermatocytes localisation in** ***Ddx4-Cre;Dcr1^fx/fx^*** **(−/−) tubules.** At P60, TUNEL-positive cells (green) are found near late prophase I spermatocytes in *Dicer1* mutant as shown by γH2AX staining of the XY body (red foci, white arrowheads in A and B). C and D show the localization of prophase I cells at the edge of the tubule (arrows) and the XY body in round spermatids (red foci, arrowheads) according the H3K9me3 immunostaining. TUNEL-positive cells localize between those two populations. E and F show the presence of few elongating spermatids stained using anti-alpha-tubulin antibody (red) which are reduced and disorganized (asterisks) in *Dicer1*-depleted germ cells tubules. Cross-sections were counterstained with DAPI.
(TIF)

Figure S3 **The early spermatogenic phase is not affected in** ***Ddx4-Cre;Dcr1^fx/fx^*** **(−/−) individuals.** H&E staining of P5 (A&B) and P10 (C&D) cross-sections from control (A&C) and −/− (B&D) seminiferous tubules. At these stages, no histological differences were observed between mutant and control testes.
(TIF)

Figure S4 **Accumulation of metaphasic-like cell population in** ***Ddx4-Cre;Dcr1 ^fx/fx^*** **(−/−)tubules.** At P60, undergoing metaphasic cells are more abundant in *Dicer1* mutant as shown by Histone H3Ser-10 phosphorylated (pH 3) positive cells (green, A and B). (C) shows a 2.5-fold increase of pH 3 positive tubules in −/− versus +/+ individuals. Cross-sections were counterstained with DAPI. Results are mean±SEM, *p<0.05, versus controls.
(TIF)

Table S1 **Frequency of** ***Ddx4-Cre;Dcr1^fx/fx^*** **obtained is lower than expected.** *Ddx4-Cre;Dcr1 ^fx/wt^* males were mated with *Dcr1^fx/fx^* females in order to produce *Ddx4-Cre;Dcr1^fx/fx^* (−/−) as well as *Dcr1^fx/fx^* (+/+) and *Ddx4-Cre;Dcr1^fx/wt^* (+/−) control littermates. The genetic background of these mice is mixed. The expected Mendelian ratio should be 25% for each genotype. Here, we obtained ∼11% of *Ddx4-Cre;Dcr1^fx/fx^*, probably due to an early recombination event in some mutant embryos resulting in embryonic death.
(TIF)

Table S2 **Table comparing reproductive and endocrine measurements between** ***Ddx4-Cre;Dcr1^fx/fx^*** **(−/−) individuals compared to** ***Dcr1^fx/fx^*** **(+/+) and** ***Ddx4-Cre;Dcr1^fx/wt^*** **(+/−) control littermates.**
(TIF)
