# Supplementary figures and images for: Dicer1 Depletion in Male Germ Cells Leads to Infertility Due to Cumulative Meiotic and Spermiogenic Defects
Source: PLoS One. 2011 Oct 5;6(10):e25241. doi: 10.1371/journal.pone.0025241 (PMC3187767; doi:10.1371/journal.pone.0025241)

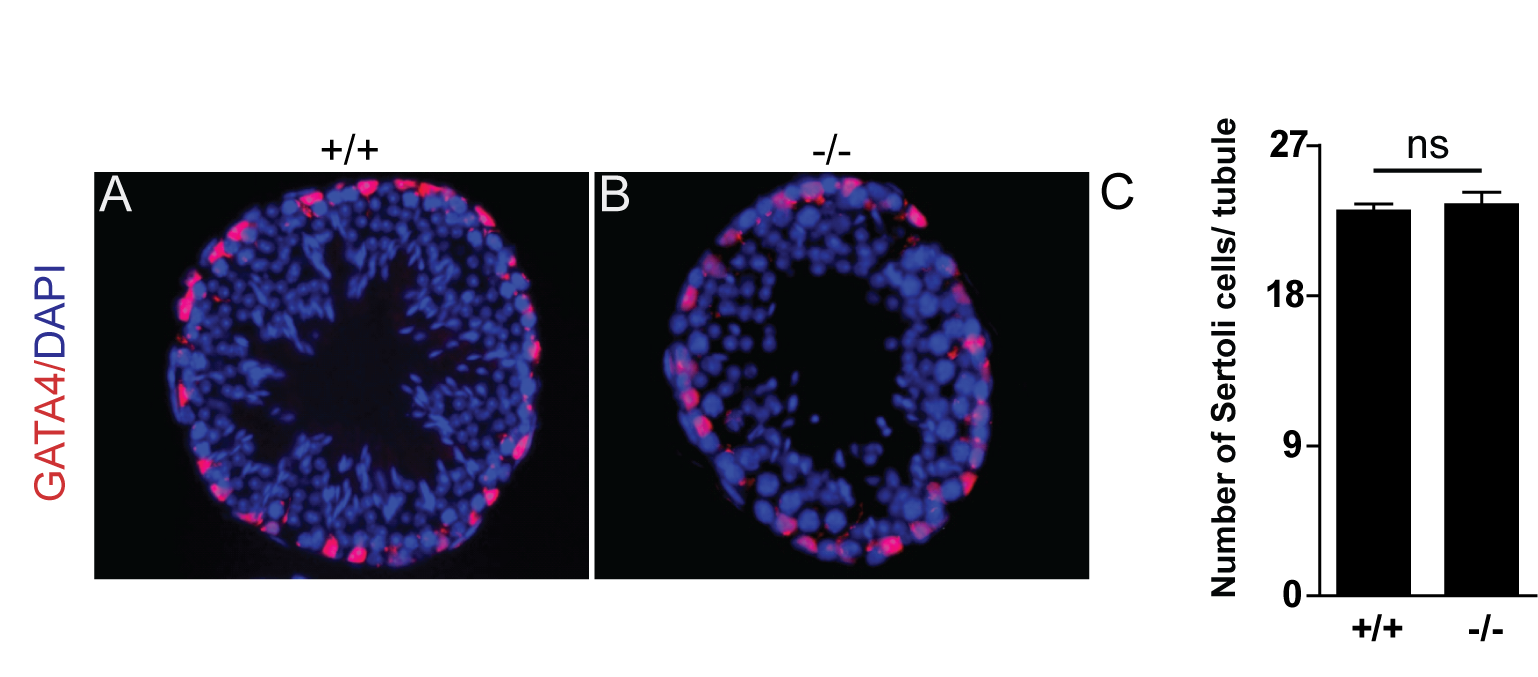

Supplement: Figure S1 — Sertoli cells organization and number are not affected in testes lacking Dicer1 in the germinal compartment. Gata-4 immunostaining (red) revealed the presence of Sertoli cells nuclei at the edge of tubules in Dcr1fx/fx (+/+) control individuals (A) as well as in Ddx4-Cre;Dcr1fx/fx mutant mice (−/−) (B), indicating that Sertoli cells organization is not affected by Dicer1 depletion in germ cells. (C) The number of Sertoli cells within seminiferous tubules is also unaffected (n = 3 animals per genotype; a minimum of 20 tubules were analyzed per individual). (TIF) [file pone.0025241.s001.tif]

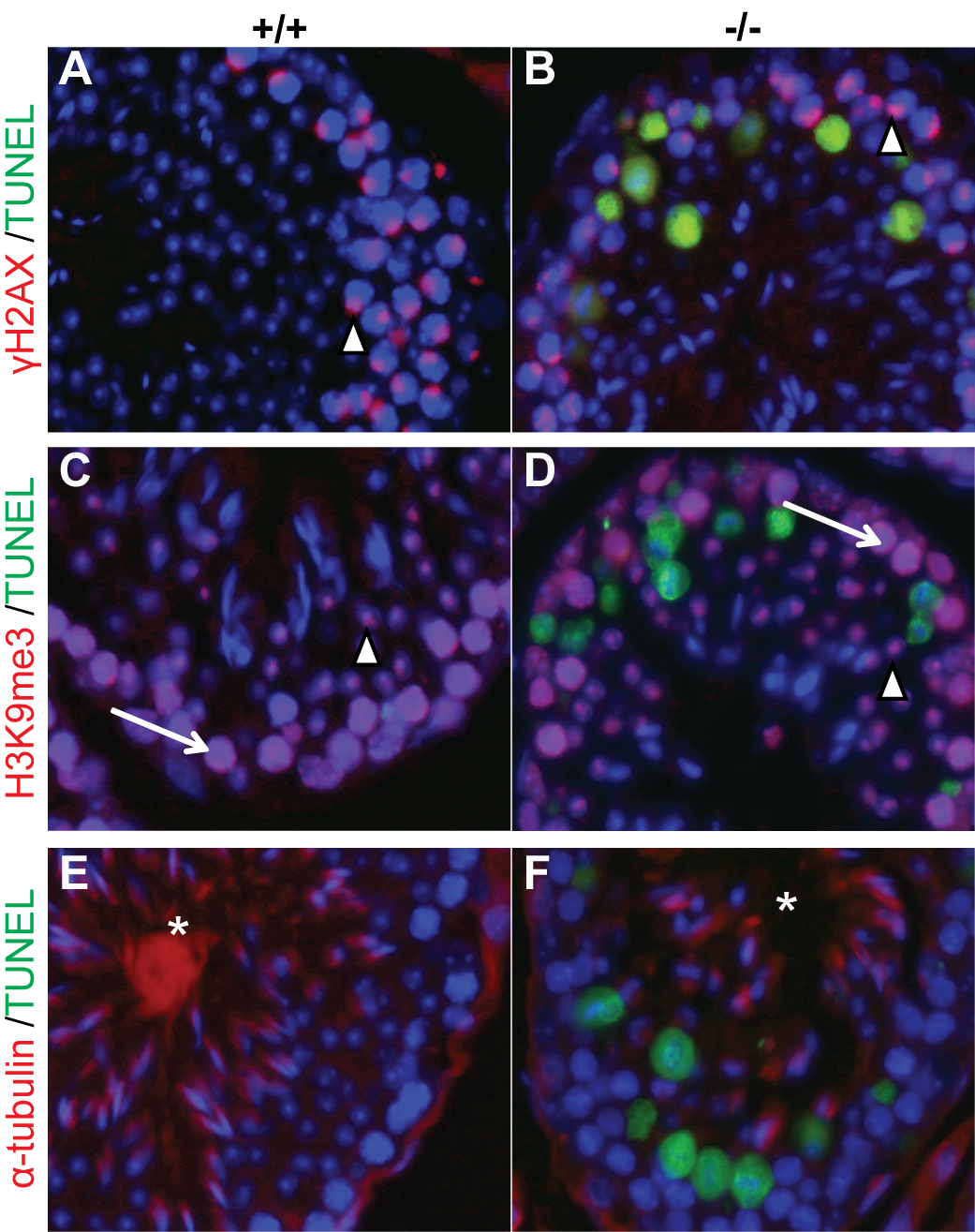

Supplement: Figure S2 — Apoptotic cell population display pachytene spermatocytes localisation in Ddx4-Cre;Dcr1fx/fx (−/−) tubules. At P60, TUNEL-positive cells (green) are found near late prophase I spermatocytes in Dicer1 mutant as shown by γH2AX staining of the XY body (red foci, white arrowheads in A and B). C and D show the localization of prophase I cells at the edge of the tubule (arrows) and the XY body in round spermatids (red foci, arrowheads) according the H3K9me3 immunostaining. TUNEL-positive cells localize between those two populations. E and F show the presence of few elongating spermatids stained using anti-alpha-tubulin antibody (red) which are reduced and disorganized (asterisks) in Dicer1-depleted germ cells tubules. Cross-sections were counterstained with DAPI. (TIF) [file pone.0025241.s002.tif]

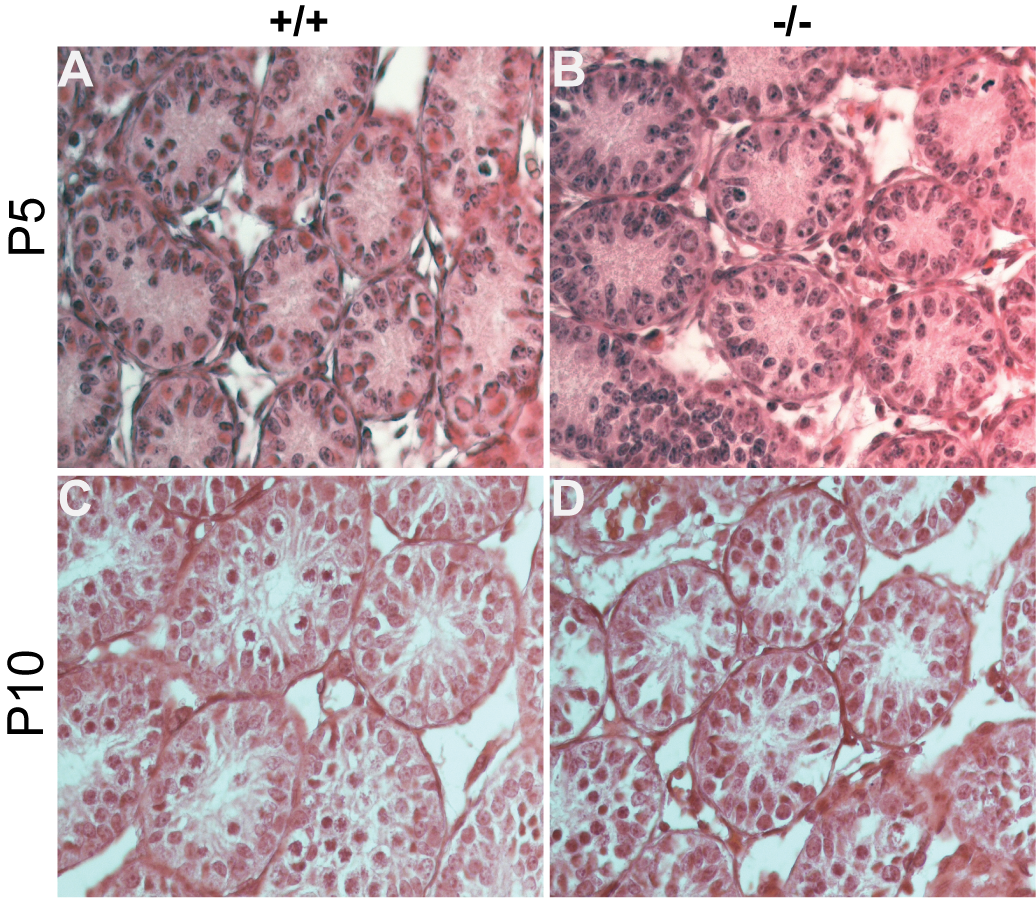

Supplement: Figure S3 — The early spermatogenic phase is not affected in Ddx4-Cre;Dcr1fx/fx (−/−) individuals. H&E staining of P5 (A&B) and P10 (C&D) cross-sections from control (A&C) and −/− (B&D) seminiferous tubules. At these stages, no histological differences were observed between mutant and control testes. (TIF) [file pone.0025241.s003.tif]

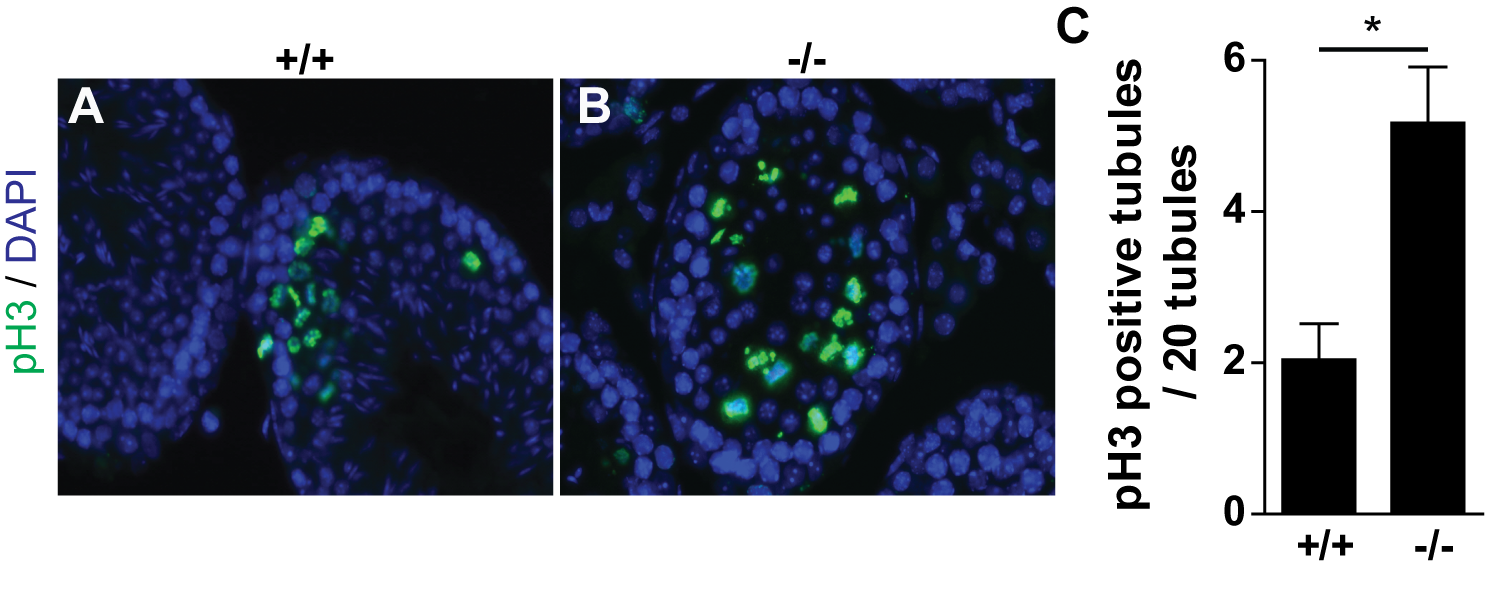

Supplement: Figure S4 — Accumulation of metaphasic-like cell population in Ddx4-Cre;Dcr1 fx/fx (−/−)tubules. At P60, undergoing metaphasic cells are more abundant in Dicer1 mutant as shown by Histone H3Ser-10 phosphorylated (pH 3) positive cells (green, A and B). (C) shows a 2.5-fold increase of pH 3 positive tubules in −/− versus +/+ individuals. Cross-sections were counterstained with DAPI. Results are mean±SEM, *p<0.05, versus controls. (TIF) [file pone.0025241.s004.tif]

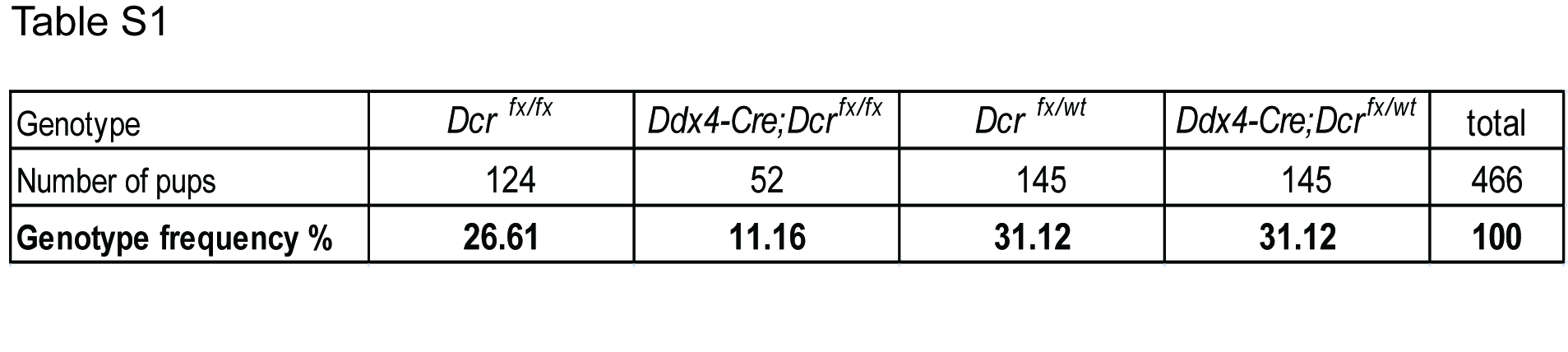

Supplement: Table S1 — Frequency of Ddx4-Cre;Dcr1fx/fx obtained is lower than expected. Ddx4-Cre;Dcr1 fx/wt males were mated with Dcr1fx/fx females in order to produce Ddx4-Cre;Dcr1fx/fx (−/−) as well as Dcr1fx/fx (+/+) and Ddx4-Cre;Dcr1fx/wt (+/−) control littermates. The genetic background of these mice is mixed. The expected Mendelian ratio should be 25% for each genotype. Here, we obtained ∼11% of Ddx4-Cre;Dcr1fx/fx, probably due to an early recombination event in some mutant embryos resulting in embryonic death. (TIF) [file pone.0025241.s005.tif]

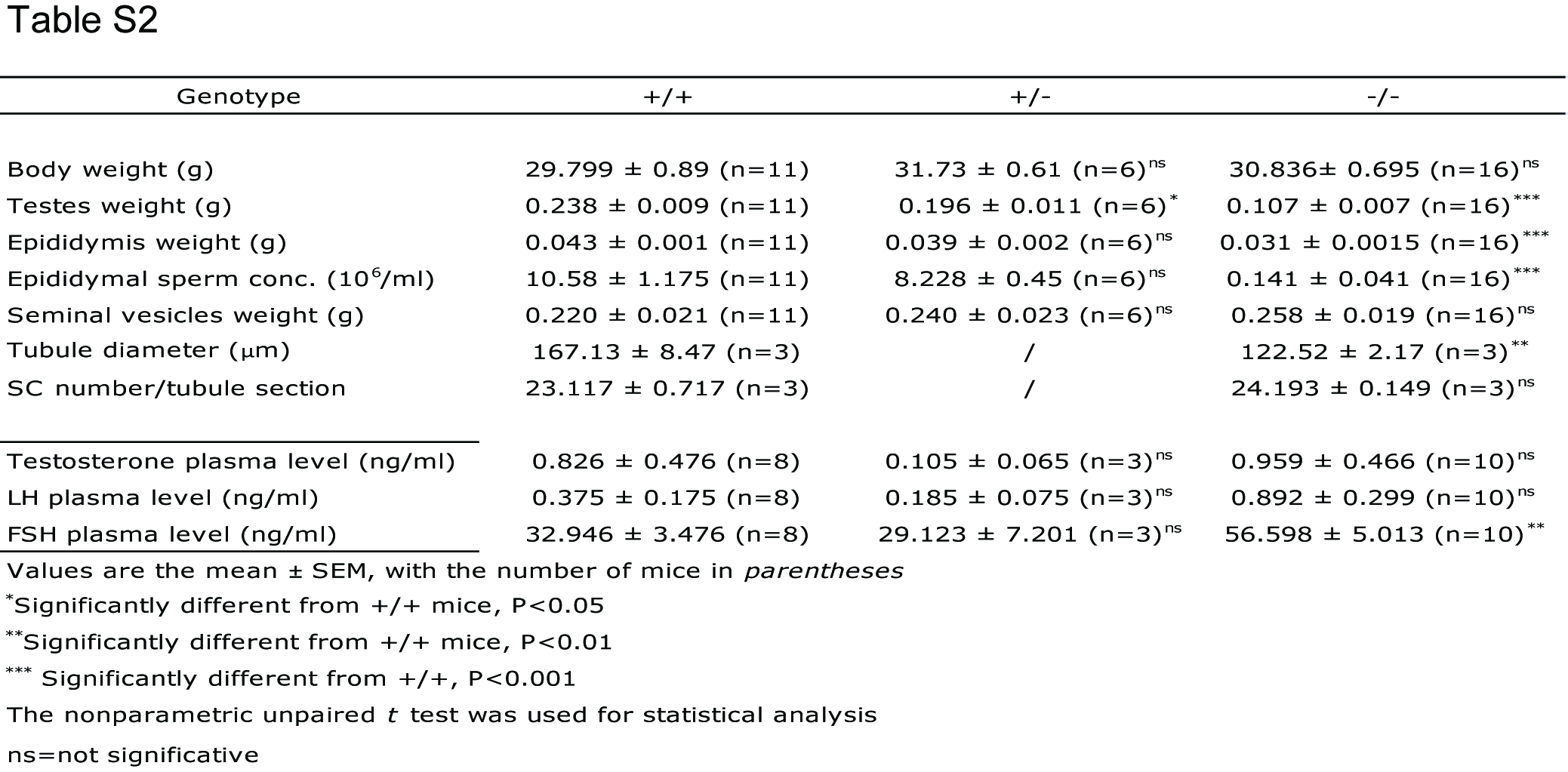

Supplement: Table S2 — Table comparing reproductive and endocrine measurements between Ddx4-Cre;Dcr1fx/fx (−/−) individuals compared to Dcr1fx/fx (+/+) and Ddx4-Cre;Dcr1fx/wt (+/−) control littermates. (TIF) [file pone.0025241.s006.tif]
